# Multi GPU parallelization of maximum likelihood expectation maximization method for digital rock tomography data

**DOI:** 10.1038/s41598-021-97833-z

**Published:** 2021-09-17

**Authors:** Jaya Prakash, Umang Agarwal, Phaneendra K. Yalavarthy

**Affiliations:** 1 Department of Instrumentation and Applied Physics, Indian Institute of Science, Bengaluru, 560 012 India; 2 Shell Technology Center, Mahadeva Kodigehalli, Bengaluru, 562 149 India; 3 Department of Computational and Data Sciences, Indian Institute of Science, Bengaluru, 560 012 India

**Keywords:** Petrology, Computational science, Electrical and electronic engineering

## Abstract

Digital rock is an emerging area of rock physics, which involves scanning reservoir rocks using X-ray micro computed tomography (XCT) scanners and using it for various petrophysical computations and evaluations. The acquired micro CT projections are used to reconstruct the X-ray attenuation maps of the rock. The image reconstruction problem can be solved by utilization of analytical (such as Feldkamp–Davis–Kress (FDK) algorithm) or iterative methods. Analytical schemes are typically computationally more efficient and hence preferred for large datasets such as digital rocks. Iterative schemes like maximum likelihood expectation maximization (MLEM) are known to generate accurate image representation over analytical scheme in limited data (and/or noisy) situations, however iterative schemes are computationally expensive. In this work, we have parallelized the forward and inverse operators used in the MLEM algorithm on multiple graphics processing units (multi-GPU) platforms. The multi-GPU implementation involves dividing the rock volumes and detector geometry into smaller modules (along with overlap regions). Each of the module was passed onto different GPU to enable computation of forward and inverse operations. We observed an acceleration of $\sim 30$ times using our multi-GPU approach compared to the multi-core CPU implementation. Further multi-GPU based MLEM obtained superior reconstruction compared to traditional FDK algorithm.

## Introduction

Digital rock is an emerging area in rock physics which aims to quantify elastic moduli, permeability, resistivity and elastic-wave velocity using high resolution representation of complex pore geometry in the rock^1^. The high-resolution representation enables us to obtain various physical properties like elasticity, transport and electrical related to the rock. These digital rock physics problems require high-resolution volumetric data to accurately resolve small pores^2^. One of the approaches to obtain these high resolution three-dimensional (3D) representation of the pore-phase and mineral-phase in the rock is through micro X-ray computed tomography ($\upmu$-XCT). Micro XCT scans enable us to measure the local X-ray attenuation (of different materials) within the scanned cylindrical rock^3,4^. To this end, typically reservoir core samples are drilled into cylindrical plugs of few millimeter diameter. These cylindrical rock geometries are then imaged using micro-CT scanners, wherein X-rays (generated by cathode-ray tubes) transmit through the rock-samples in a cone-beam configuration and are detected using a two-dimensional flat panel detector/CCD-camera in a trans-illumination mode. The sample is then rotated to obtain tomographic measurements (projections) at various angles.

The obtained tomographic measurements are used to reconstruct three-dimensional volumetric information of the scanned rock^5^. The aim of reconstruction process is to convert the information contained in acquired projections to X-ray attenuation coefficient maps which represent different phases of the rock geometry such as pore, grain, clay, minerals, and their respective boundaries^2^. The 3D reconstruction is typically performed using direct backprojection (analytical) methods, like Feldkamp–Davis–Kress (FDK) algorithm due to its lower computational footprint^6^. However, analytical methods are known to generate 3D volumes that are not quantitative in nature^7^. Recent emphasis has been on developing iterative methods, which have demonstrated potential in resolving the 3D rock volumes with higher quantitative accuracy compared to analytical methods^7^. These methods also help to incorporate prior information for improving the reconstruction quality and for dynamic tomography of time resolved processes in porous media. Iterative methods tend to be computationally expensive compared to analytical methods, making them less attractive while working with large datasets^5,8^. Iterative reconstruction techniques involve matching the measured tomographic data with the signals predicted by the model in an iterative fashion^8^. Hence iterative methods require repeated computation of forward and inverse operators, making it computationally challenging for large dataset that commonly arise in digital rock analysis.

Iterative reconstruction in computed tomography is performed by algebraic schemes (simultaneous algebraic reconstruction technique (SART), simultaneous iterative reconstruction technique (SIRT)) and statistical schemes (maximum likelihood expectation maximization (MLEM), ordered subset expectation maximization (OSEM)). Earlier works in digital rock have deployed SART and reported high accuracy compared to the FDK approach^7^. However the SART reconstruction was restricted to volumes of size $1024 \times 1024 \times 1024$ (with only 400 projections of $1024 \times 1024$) to allow practical computation on multi-core CPU^7^. The main aim of this work is to enable iterative reconstruction with large-scale digital rock data. Earlier works have parallelized iterative reconstruction algorithms on graphics processing units (GPU), wherein the matrix operations was parallelized on GPU environments^9^. Other approach included developing multi-threaded approach for each ray to accelerate forward projection operations on GPU environment^10^, which is different from the block based decomposition proposed in this work. Further three-dimensional ordered sub-set expectation maximization approach was proposed in the context of PET^11^, however this approach was working with small imaging volumes. A detailed review on developments of GPU based parallelization for image reconstruction problems can be found in Ref.^12^. Since SART scheme was developed on single-GPU platform, performing reconstruction at higher resolution is prohibited due to limited available on-board memory on single GPU. Note that MLEM approach is statistical in nature and used routinely in positron emission tomography (PET) as opposed to SART where the data is typically noisy. Further, building a system matrix for large-scale three-dimensional problems is not feasible due to the memory overhead associated with storing the matrix, hence our work parallelized the MLEM reconstruction using forward/inverse operators. In this work, we have utilized MLEM approach for resolving three-dimensional volumes of rock samples. Further the MLEM scheme was parallelized on multiple graphics processing units (multi-GPU) platforms. Specifically, the most time-consuming operations in the MLEM algorithm was the forward and inverse operators, these operators were parallelized on multi-GPU architecture, to provide much needed acceleration for MLEM algorithm.

The paper is organized by initially explaining the materials and methods developed. Next section elaborates on the results pertaining to the comparison of FDK and MLEM approaches. This is followed by a discussion section of the observed results, lastly the manuscript briefly explains the conclusions and possible future work.

## Materials and methods

### Maximum likelihood expectation maximization

MLEM approach involves two operations, namely forward projection (forward operator) and backprojection (inverse operator).

#### Forward projection operation

Modelling the penetration of X-rays through the rock using cone-beam configuration can be achieved using Radon operator. Let *f*(*x*, *y*, *z*) be the object being scanned and $P(\theta ,u,v)$ be the recorded projections at the flat panel detector at an angle $\theta$. The forward projection is then defined as^5,6^,

1 $$\begin{aligned} P(\theta , u, v) = \int _0^\infty f \left(\mathbf{a} (\theta ) + t \left[ \frac{u\mathbf{e_u} (\theta ) + d \mathbf{e_w} (\theta ) + v \mathbf{e_v} (\theta )}{\sqrt{u^2+d^2+v^2}} \right] \right) dt \end{aligned}$$

where *u*, *w*, *v* are local detector coordinates with unit vectors given as $\mathbf{e_u}(\theta ) =[-sin(\theta ), \ cos(\theta ), \ 0]^T$, $\mathbf{e_w}(\theta ) =[-cos(\theta ), \ -sin(\theta ), \ 0]^T$ and $\mathbf{e_v}(\theta ) =[0, \ 0, \ 1]^T$. Note that the unit vector $\mathbf{e_w}$ represents the axis from source to the center of the detector and the unit vectors $\mathbf{e_u}$ and $\mathbf{e_v}$ spans the X-ray detector array. Further $\mathbf{a} (\theta ) = [R_0 cos(\theta ), \ R_0 sin(\theta ), \ 0]$ with $R_0$ in the radius of source trajectory (equivalent to $\frac{d}{2}$, where *d* indicates the source to detector distance).

#### Backprojection operation—FDK algorithm

The FDK algorithm backprojects the detected 2D projections onto 3D volume along with the scaling factors (which are based on source to detector distance and source to object distance). The 2D projection gets backprojected onto the 3D volume. The overall FDK based reconstruction can be written as^5,6^,

2 $$\begin{aligned} f_{FDK}(r,\phi ) = \frac{1}{4\pi ^2} Re \oint d\Phi \frac{d^2}{(d+\rho .\mathbf{e_w})^2} \int _{-\infty }^{\infty } dY \frac{d}{\sqrt{d^2 +Y^2}} P (Y,\Phi ) h_H(Y'-Y) \end{aligned}$$

where $f_{FDK}(r,\phi )$ is the reconstruction volume in image space at a point indicated as $(r,\phi )$, *P* is the projection at an angle $\Phi$. $h_H(Y'-Y)$ indicates the Hilbert kernel^13^. $\rho$ is the vector starting from the origin in the mid-plane to the reconstruction point.

#### Maximum likelihood expectation maximization (MLEM)

MLEM approach involves maximizing the log likelihood function of Poisson statistics. MLEM can provide accurate quantitative reconstructions compared to analytical techniques in the limited data situations. The formulation for MLEM type approach can be written as^8^,

3 $$\begin{aligned} X_{MLEM}^k = \frac{X_{MLEM}^{k-1}}{\sum _{i=1}^n a_{ij}} \left[ \sum _{i=1}^n \frac{P_i}{\sum _{j=1}^n a_{ij}X_{MLEM}^{k-1}} a_{ij} \right] \end{aligned}$$

where $X_{MLEM}^k$ is MLEM reconstruction output at *k*th iteration and $X_{MLEM}^{k-1}$ is the MLEM reconstruction output at $(k-1)$th iteration. Projections are represented using $P_i$, $a_{ij}$ indicates the entries of system matrix at location *i* and *j*. The above equation can be elaborated as,

4 $$\begin{aligned} (image)^{k} = (image)^{k-1} \times Normalization \left[ \ BP \left[ \frac{Experimental\ Projection}{FP(image)^{k-1}}\right] \right] \end{aligned}$$

where *FP* and *BP* indicates the forward projection and backprojection operations explained in earlier section wherein the variables *image* and *Experimental Projection* are in three dimensions while performing the forward and back projection.

#### Multi-GPU based MLEM

As stated earlier, our work parallelized the MLEM algorithm using the forward and inverse operators as opposed to using the system matrix approach. The forward and inverse operators are implemented based on linear interpolation operations between the projection space and the imaging volume rather than using a matrix operator^13^. Note that building the system matrix is not feasible for digital rock problems, because the number of measurements are $1536 \times 1536 \times 1600$ and the number of unknowns are $1536 \times 1536 \times 1536$ resulting in a matrix size of 1,887,436,800 $\times$ 3,623,878,656 which is impractical to implement on currently available GPU platforms. Therefore, our work parallelized the MLEM algorithm by deploying forward and inverse operators on multi-GPU platforms.

**Figure 1:**
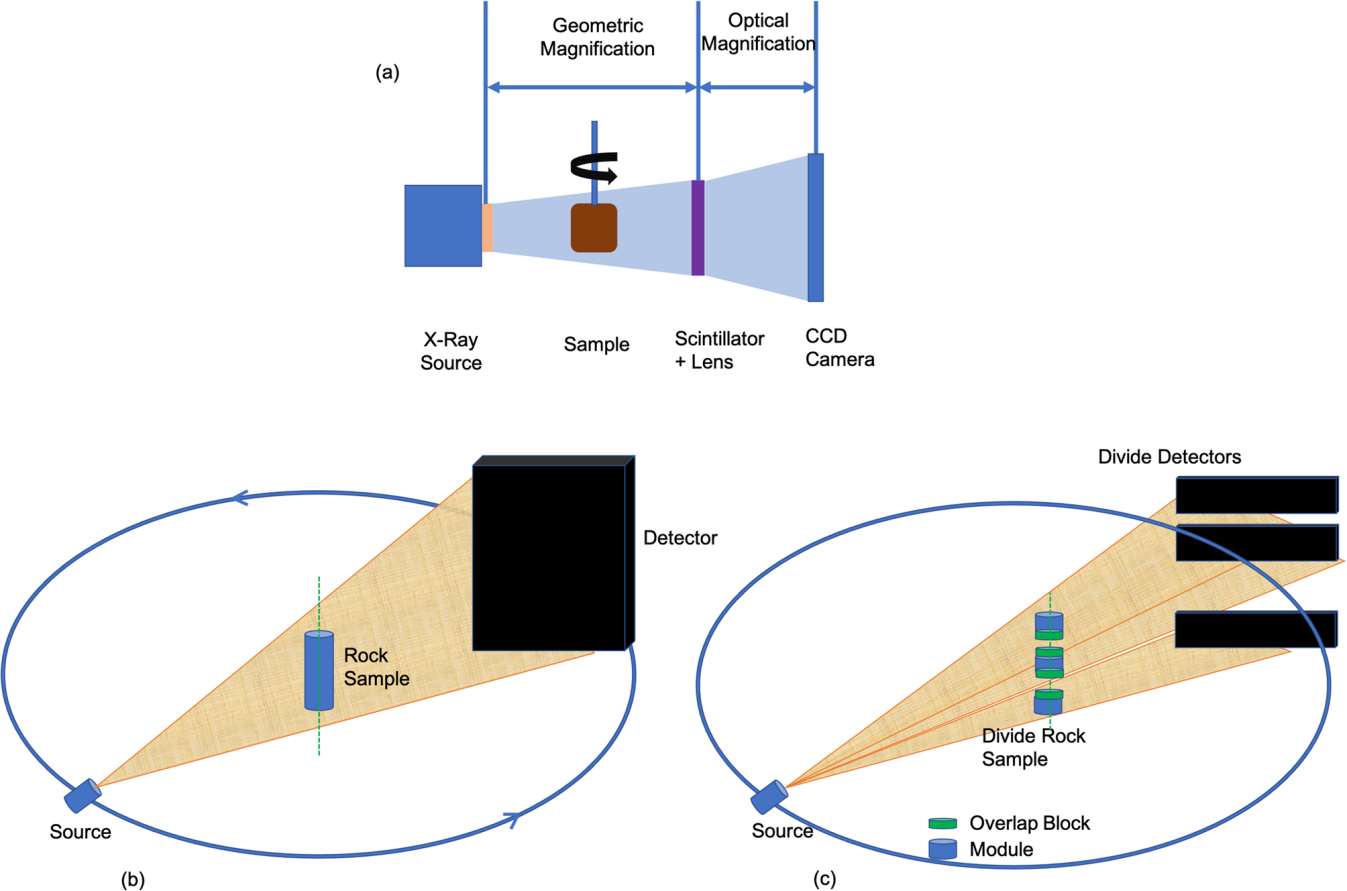
(**a**) The illustration of the X-ray micro-CT system configuration for data acquisition. (**b**) The schematic used for acquiring the projections using cone-beam CT configuration. (**c**) The proposed scheme of dividing the rock and the detector array while performing the parallel MLEM reconstruction.

Figure 1 shows the system used for acquiring the digital rock projection data. Figure 1b indicates the tomographic acquisition of the projections from different angles, where the rock sample will be rotated. As can be seen from Fig. 1c, the cylindrical rock was divided into multiple smaller blocks (which we call as module) along with a small overlap region; such a framework is considered to include accurate boundary conditions while implementing the forward and inverse operators during modularization on multiple GPUs. This overlap region was considered both at the top and the bottom of the module; except near the boundaries. The overlap block was indicated in green colour in Fig. 1c and modules of the rock were indicated in the blue colour. These blocks (module + overlap block) were then transferred to different GPU’s to enable computation of forward and inverse operations. Note that the detector array corresponding only to the size of the module was updated by each GPU while performing the forward operation, similarly only the module in the rock sample is updated while performing the inverse operation.

The mathematical equations used for forward operation is

5 $$\begin{aligned} P(\theta , u_{det}, v_{det})_{GPU} = \int _0^\infty f_{module} \left(\mathbf{a} (\theta ) + t \left[ \frac{u_{det} \mathbf{e_u} (\theta ) + d \mathbf{e_w} (\theta ) + v_{det} \mathbf{e_v} (\theta )}{\sqrt{u_{det}^2+d^2+v_{det}^2}} \right] \right) dt \end{aligned}$$

where $u_{det}, v_{det}$ indicates the local coordinate representation of the detector array on that particular GPU (indices corresponding to detector array on the GPU), and $f_{module}$ represents the object corresponding to the module along with overlap region. Further $P(\theta , u_{det}, v_{det})_{GPU}$ indicates the projections that was updated corresponding to the indices represented by $u_{det}, v_{det}$ on a particular GPU. All the projections from different GPU’s are accumulated to generate $P(\theta , u , v)$. The parallelization of the inverse operation is given as

6 $$\begin{aligned} f_{FDK}(r_{module},\phi _{module})_{GPU} = \frac{1}{4\pi ^2} Re \oint d\Phi \frac{d^2}{(d+\rho .\mathbf{e_w})^2} \int _{-\infty }^{\infty } dY_{det} \frac{d}{\sqrt{d^2 +Y_{det}^2}} P (Y_{det},\Phi ) h_H(Y_{det}'-Y_{det}) \end{aligned}$$

where $P(Y_{det},\Phi )$ indicates the projections on the modularized detector array at a projection angle $\Phi$ on a particular GPU, which is integrated over all the angles and the backprojected result was stored as $f_{FDK}(r_{module},\phi _{module})_{GPU}$ corresponding to locations indicated by $(r_{module},\phi _{module})$ on that particular GPU. All the backprojected values from different GPUs were accumulated to generate $f_{FDK}(r,\phi )$. Again, the forward and inverse operations (Eqs. 5 and 6) are implemented using linear interpolation operations. Note that these values from forward and backprojection were transferred to the CPU, where the MLEM algorithm was executed as indicated in Eq. (4) on the multi-core CPU. The details regarding the data flow from CPU to multi-GPU for both forward and inverse operation is given in the supplementary. The MLEM algorithm was performed on a multi-core CPU (Intel Xeon E5-2695v3 2.3 GHz with total 56 cores, 512 GB RAM) and a multi-GPU architecture (Intel Xeon Silver-4110 CPU with 2.10 GHz and 16 cores, 256 GB RAM with 16 Nvidia GK210GL Tesla K80 GPU cards).

### Image acquisition protocol

To obtain the cone-beam CT (CBCT) data of the rock sample, rock samples were scanned with the Xradia 520 Versa Zeiss micro-CT scanner. A CCD based detector was used to capture the projections obtained by rotating the rock sample over 3200 viewing angles for a 360 degrees rotation to capture 3201 projections. The detector array was having a configuration of $2020 \times 2020$ pixels (with physical size as $6.464 \times 6.464$ mm) and each projection was acquired with an exposure time of about 2 s. The rock sample was a Bentheimer sandstone, which was drilled to a 4 mm diameter resulting in a resolution of about 2 microns for an imaging volume of size $2020 \times 2020 \times 2020$ voxels. This rock was glued onto the sample holder which was rotated for acquiring the projections. The X-ray source was having a tube voltage of about 80 kV (operating in a possible range from 50 to 160 kV). The source to detector distance was set as 26.07 mm and the source to object distance was set to 16.06 mm. From this acquired dataset we used $1536 \times 1536$ projection data and 1600 projections (which are within the acceptable limits of sampling requirement for analytical reconstruction^13^) to reconstruct the three dimensional imaging volume of $1536 \times 1536 \times 1536$ at 2 micron resolution using MLEM algorithm.

## Results

**Figure 2:**
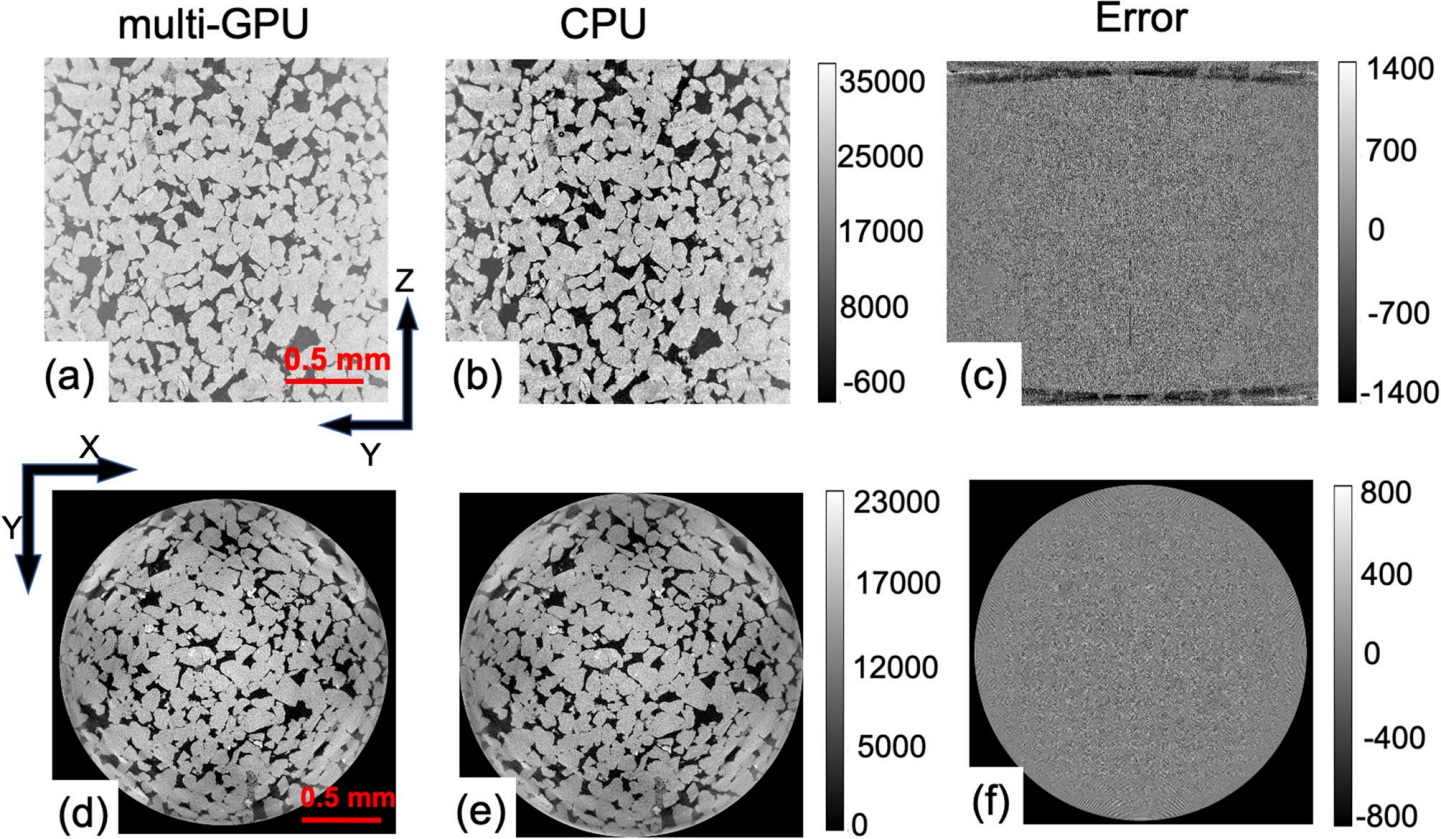
Comparison of MLEM reconstruction performance using multi-GPU and multi-core CPU architectures: (**a**) Reconstructed image using multi-GPU architecture along the Y–Z plane having X = 0. (**b**) Reconstructed image using multi-core CPU architecture along the Y–Z plane having X = 0. (**c**) The difference image of the multi-core CPU and multi-GPU implementations along the Y–Z plane having X = 0. (d) Reconstructed image using multi-GPU architecture along the X–Y plane having Z = 0. (**e**) Reconstructed image using multi-core CPU architecture along the X-Y plane having Z = 0. (**f**) The difference image between the multi-core CPU and multi-GPU implementations along the X–Y plane having Z = 0.

The CT projection data acquired from the Zeiss scanner was used to reconstruct the three-dimensional rock volume using multi-GPU and multi-core CPU architectures. The results pertaining to multi-GPU implementation along the Y–Z and X–Y planes are indicated in Figs. 2a,d, respectively. CT reconstructed images using the multi-core CPU implementation was shown in Figs. 2b,e, respectively. The difference image obtained by subtracting the multi-GPU CT image and multi-core CPU CT image along the Y–Z plane and X–Y plane is shown in Figs. 2c,f, respectively. Overall Fig. 2 indicates that the error between the CPU and multi-GPU implementation is very small, specifically the error seems to be high near the boundary of the rock, which might be due to the Dirichlet boundary condition (i.e. not having any overlap rock volumes on both sides of the rock boundaries) in the multi-GPU implementation. As shown in the data flow graph, only small parts of volume/projections (along with overlap block) are sent to each GPU which are used to compute the forward and inverse operations, as opposed to CPU which uses the entire volume/projection space to compute the forward and inverse operators. As a result, we expect small amount of errors between CPU and multi-GPU implementations.

**Figure 3:**
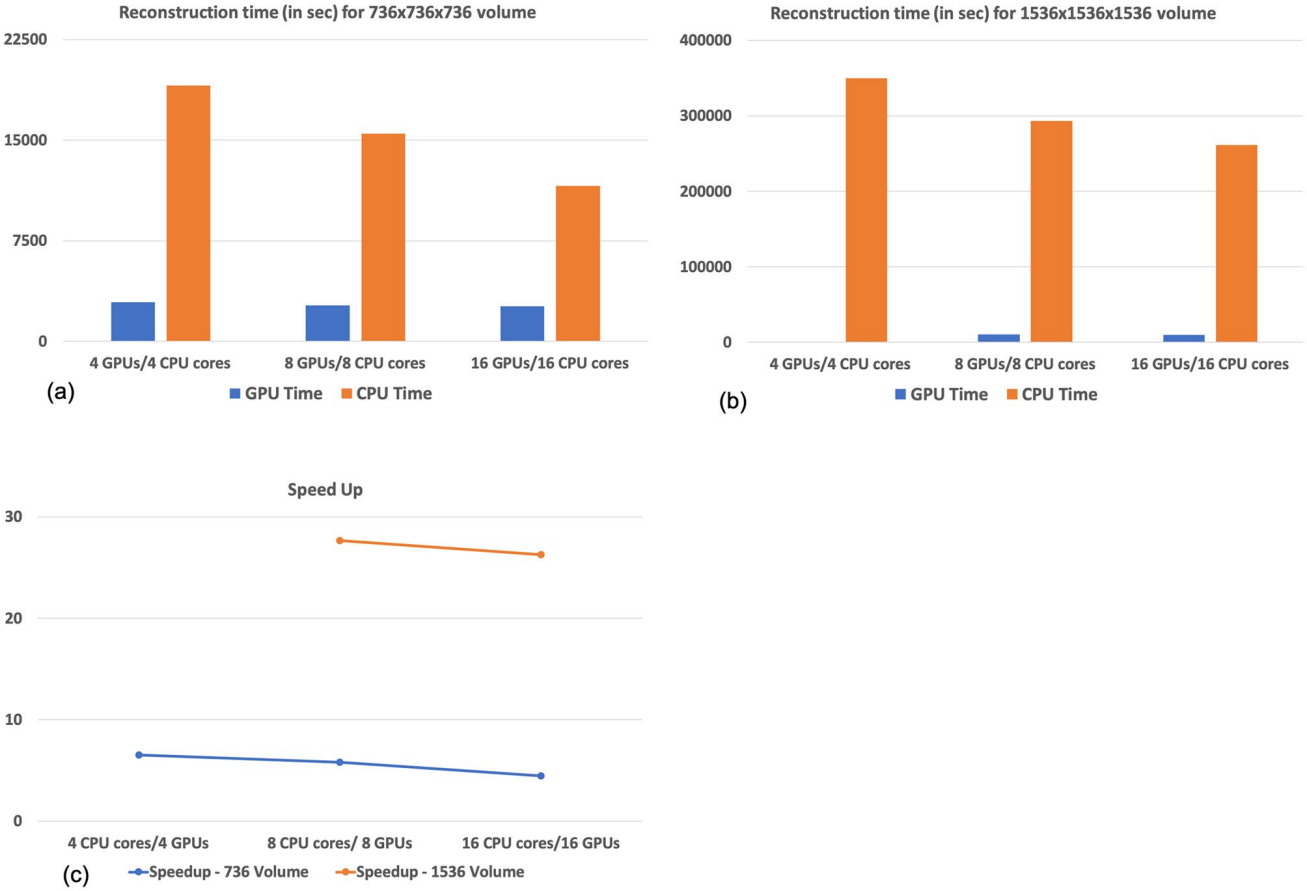
Comparison of the computational performance between the multi-GPU and multi-core CPU implementations. (**a**) Reconstruction time (in s) corresponding to multi-GPU and CPU implementation for reconstructing a $736 \times 736 \times 736$ volume, (**b**) reconstruction time (in s) corresponding to multi-GPU and CPU implementation for reconstructing a $1536 \times 1536 \times 1536$ volume, (**c**) the speed up achieved using the multi-GPU implementation over the CPU implementation with different rock volume sizes.

The computational comparison for single iteration of MLEM, which involves computing the forward and inverse operations was shown in Fig. 3. The computational time with multi-GPU and multi-core CPU implementations corresponding to reconstructing a $736 \times 736 \times 736$ volume was shown in Fig. 3a. Similarly, the computational time while reconstructing a $1536 \times 1536 \times 1536$ volume was shown in Fig. 3b. The speed-up achieved by using a multi-GPU architecture compared to multi-core CPU was shown in Fig. 3c. As can be seen, the multi-GPU implementation is scalable with the problem size, the speed-up increased from $\sim 7$ times using $736 \times 736 \times 736$ volume to $\sim 28$ times using the $1536 \times 1536 \times 1536$ volume. This kind of scalable implementation is highly desirable especially in the context of digital rock, wherein large imaging volumes are involved to achieve high-resolution. For the case of eight GPUs and $1536 \times 1563 \times 1536$ volume size, module was set to 192 slices and overlap block of 48 ($\frac{1}{4}{th}$ of the module size) slices on both sides of the rock. Similarly for the case of 16 GPUs, the module was set to 96 slices and overlap block of 24 slices on both sides of the rock. However, for the case of 4 GPUs, implementation using $1536 \times 1536 \times 1536$ volume was not feasible on multi-GPU due to the limited available on-board GPU memory, which is currently limited to 32 GB (Nvidia Tesla GV100).

**Figure 4:**
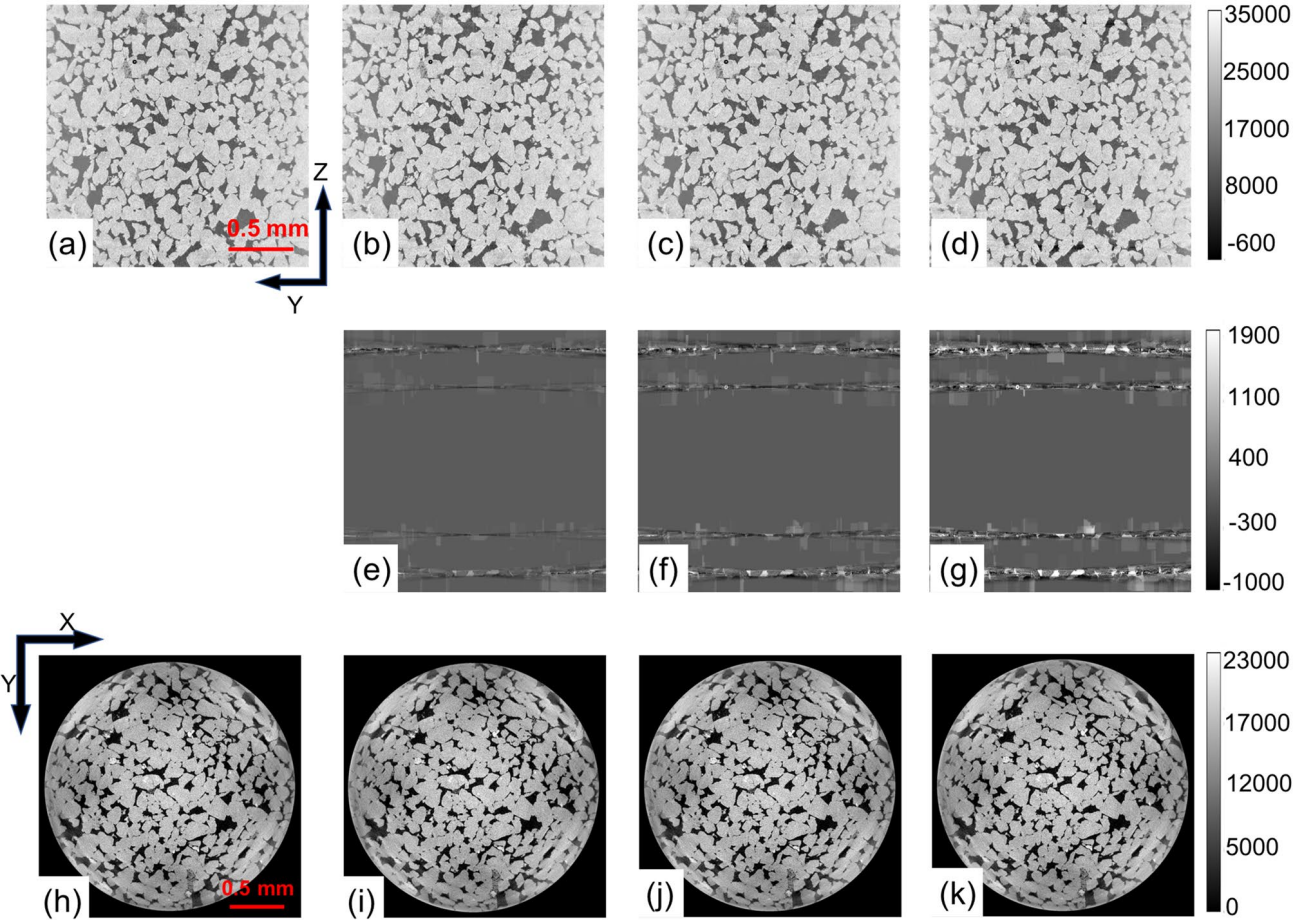
Reconstruction results obtained by varying the overlap size in the multi-GPU implementation. Reconstruction results corresponding to the Y–Z plane at X = 0 for $1536 \times 1536 \times 1536$ volume using overlap size as (**a**) 48 (**b**) 39 (**c**) 32 (**d**) 24. The difference image (on the Y–Z plane at X = 0) between (**e**) overlap size as 48 and 39 (**f**) overlap size as 48 and 32 (**g**) overlap size as 48 and 24. Reconstruction results corresponding to the X–Y plane at Z = 0 for $1536 \times 1536 \times 1536$ volume using overlap size as (**h**) 48 (**i**) 39 (**j**) 32 (**k**) 24.

Next, we also investigated the choice of overlap size on the quality of the reconstructed rock images. To this end, MLEM reconstructions (with $1536 \times 1536 \times 1536$ volume) were performed by varying the overlap block size (48, $\frac{1}{4}{th}$ of module size; 39, $\frac{1}{5}{th}$ of the module size; 32, $\frac{1}{6}{th}$ of the module size; 24, $\frac{1}{8}{th}$ of the module size). The reconstructions corresponding Y–Z plane at X = 0 corresponding to overlap sizes as $\frac{1}{4}{th}$, $\frac{1}{5}{th}$, $\frac{1}{6}{th}$, $\frac{1}{8}{th}$ of the module size is shown in figs. 4a–d. The difference images between the overlap size as 48 and 39 was shown in Fig. 4e. The difference images between the overlap size as 48 and 32 is shown in Fig. 4f. The difference images between the overlap size as 48 and 24 is shown in Fig. 4g. The difference images indicate that the error tends to arise at the edges of each module, which is expected. Further the errors tend be higher with lower overlap size and the error reduces as the overlap size is increased. Lastly, the reconstructions corresponding X–Y plane at Z = 0 corresponding to overlap sizes as 48, 39, 32, and 24 is shown in figs. 4h–k. The errors at the central slice was found to be negligible (hence was not shown here).

**Figure 5:**
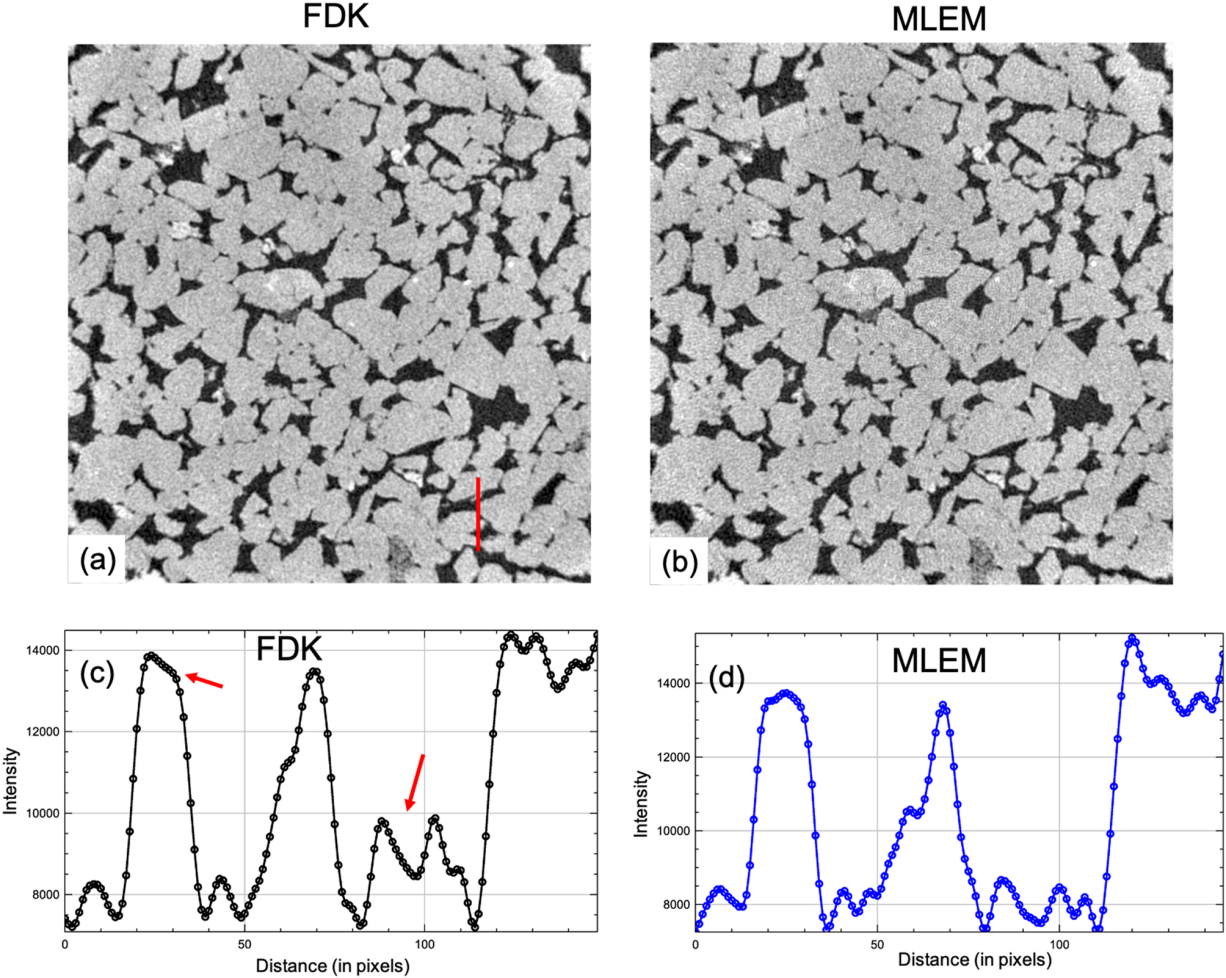
Comparison of FDK and MLEM reconstruction. (**a**) Reconstructed image obtained using FDK algorithm. (**b**) Reconstructed image obtained using multi-GPU MLEM based algorithm. (**c**) Line profile along the red line indicated in (**a**) using the FDK algorithm. (**d**) Line profile along the red line indicated in (**a**) using the MLEM algorithm.

Lastly, the FDK reconstruction was compared with the MLEM reconstruction. The reconstructed central slice obtained using the FDK and MLEM approach is shown in figs. 5a,b, respectively. Here the MLEM algorithm was run for about 6 iterations and only 800 projections were considered for both the FDK and MLEM reconstructions. The line plot along the red line in Fig. 5a for the FDK and MLEM approaches are given in figs. 5c,d, respectively. The red arrow in the line plot (Fig. 5c) indicates that the FDK approach was not able to reconstruct the intensities within the grain homogeneously i.e. the grain intensity near the 25*th* pixel is not having a step like response using FDK while a step like response was retained using MLEM algorithm. Further the reconstructed intensities from the pore regions (around 100*th* pixel) seems to be amplified using the FDK approach compared to the MLEM approach (which is indicated again by the red arrow in Fig. 5c). Hence it can be concluded that the MLEM algorithm has the ability to provide superior reconstruction compared to FDK approach in limited data setting.

## Discussion

Earlier works have developed analytical solvers like FDK on multi-GPU framework^14^. Further Tomographic Iterative GPU-based Reconstruction (TIGRE) toolbox was also developed to run iterative reconstruction on MATLAB-based CUDA solvers^9^. Note that TIGRE toolbox was capable of running these algorithms on single GPU cards^9^, which may not be helpful to run large-scale problems relevant to digital rock platforms. Obtaining high-resolution reconstruction with sub-micron resolution is the need of the hour for digital rock platforms to enable geologists to accurately resolve pore-scale distribution which plays crucial role in mercury injection capillary pressure and other transport properties^15,16^. However, with the current available hardware, this would not be possible requiring more advances in GPU hardware to enable increased on-board GPU memory for performing these memory-intensive computations. It is important to note that the proposed method is scalable, since we were able to run multi-GPU accelerated MLEM algorithm on rocks having different sizes i.e. $736 \times 736 \times 736$ and $1536 \times 1536 \times 1536$. Hence, we expect, easy translation of the proposed methodology with better hardware capabilities thereby enabling the much-needed acceleration for large datasets arising in digital rock. The results in Fig. 3 suggest that the optimal solution would involve reconstructing maximum possible volume on a single GPU card or minimizing the number of subdivisions, as this will ensure minimum latency. The scalability is limited due to the memory transfer between CPU and GPU taking majority of the time. However, using single GPU card would be limited by the hardware architecture and the volume of the geometry one wish to reconstruct owing to finite memory on a single GPU card (much smaller than regular CPU memory). Note that multi-GPU platforms are necessary due to GPU memory for bigger digital rock volumes even if the resulting parallel efficiency is limited.

Lastly, based on our assessment, we found that the higher the overlap volume size, the better is the reconstruction quality compared to the multi-core CPU implementation. This fact brings a tradeoff between the GPU memory (based on module size + overlap size) and the reconstruction accuracy that could be achieved. Our analysis indicated that having an overlap size of approximately equal to $\frac{1}{4}{th}$ of the module size was enough to obtain the reconstruction accuracy similar to that of multi-core CPU implementation. Further the variations in computational time with different overlap volume size was found to be insignificant. Since the forward-projection and back-projection operations have been parallelized on multi-GPU architectures, advanced reconstruction schemes based on compressive sensing, total-variation, half-quadratic regularization schemes can be explored in the context of digital rock^17–19^. Further the CT reconstruction seems to have beam-hardening effects (figs. 2d,e), which could be potentially mitigated by deploying these advanced reconstruction methods.

It is also important to note that per core cost of GPU is much lesser compared to CPU. Overall given the cost effectiveness of GPU, it is always desirable to implement the advanced reconstruction algorithms especially involving large high-resolution CT data, in scenarios like digital rock, in the multi-GPU environment both from cost-effectiveness as well as computational time. This work has shown the advantage of multi-GPU environment and have paved a way for deploying advanced reconstruction methods for digital rock. As part of future work, the multi-GPU codes could be further accelerated by implementing this methodology in light-weight programming languages like C alongside CUDA, and implementing custom-made kernels to parallelize forward projection and back projection operations. Note that the main bottleneck is scalability to larger volumes with minimum latency, which can be addressed by implementing parallelization operations using C programming in CUDA framework.

## Conclusion

Digital rock involves reconstructing large three-dimensional imaging volumes of rock samples at high-resolution which influence evaluation of various petrophysical properties. These large CT datasets are typically reconstructed using analytical schemes as model based iterative solvers are computationally intractable due to huge computational complexity associated with their implementation on multi-core CPUs. In this work, we have parallelized the MLEM algorithm on multi-GPU architecture to enable much needed scalability for using iterative reconstruction algorithms for digital rock implementation. Each iteration of MLEM involves performing one forward and one inverse operation which are the main computational bottlenecks, hence we have parallelized these operations on multi-GPU platform. Specifically, the rock volume and detector array were divided into many modules (along with overlap region) and each module was transferred to different GPU cards to enable accelerated computation of forward and back-projection operations. The operations on each card are load balanced reducing any latency in their implementation. The results indicated that the proposed multi-GPU implementation was approximately thirty times faster than multi-core CPU implementation. Further the error between multi-GPU and multi-core CPU implementation was minimal, making the multi-GPU implementation attractive for further investigation of iterative methods for digital rock applications.

## Supplementary information

Supplementary Information.

## Data Availability

The codes can be obtained from the corresponding author at reasonable request.
