# Supplementary material for: Multi GPU parallelization of maximum likelihood expectation maximization method for digital rock tomography data
Source: Sci Rep. 2021 Sep 17;11:18536. doi: 10.1038/s41598-021-97833-z (PMC8448866; doi:10.1038/s41598-021-97833-z)
Supplement: Supplementary file 1 — Supplementary Information. [file 41598_2021_97833_MOESM1_ESM.pdf]

SUPPLEMENTARY FOR: Multi GPU parallelization of maximum likelihood expectation maximization method for digital rock tomography data

Jaya Prakash[1,†,*], **Umang Agarwal**[2,†], **Phaneendra K. Yalavarthy**[3]
[1]Department of Instrumentation and Applied Physics, Indian Institute of Science, Bengaluru - 560 012.
[2]Shell Technology Center, Mahadeva Kodigehalli, Bengaluru - 562 149.
[3]Department of Computational and Data Sciences, Indian Institute of Science, Bengaluru - 560 012.
[*]Corresponding author: jayap@iisc.ac.in
[†]Equal Contribution

This document provides the supplementary information for the original manuscript.

1 Data Flow

Supplementary Figs S1 and S2 indicates the data flow from CPU to multi-GPU while computing the forward and inverse operators.

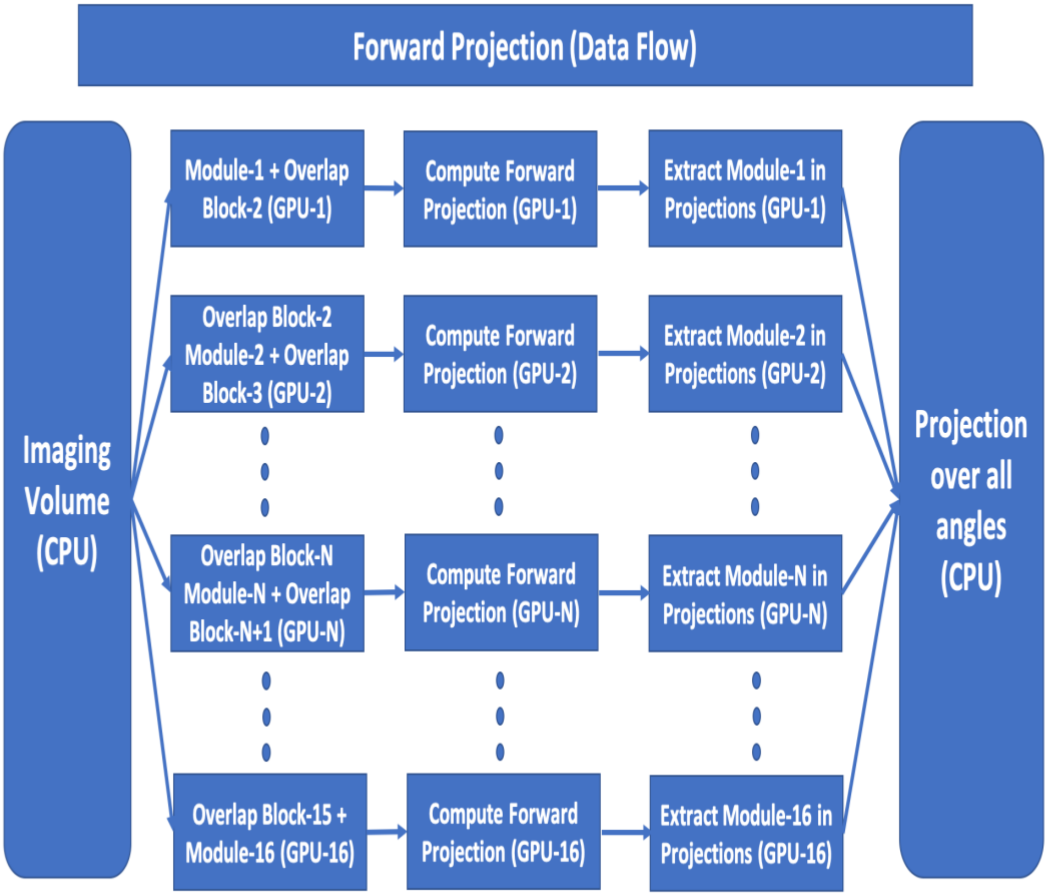

Supplementary Fig. S1: Data Flow from CPU to multi-GPU while computing the forward projection

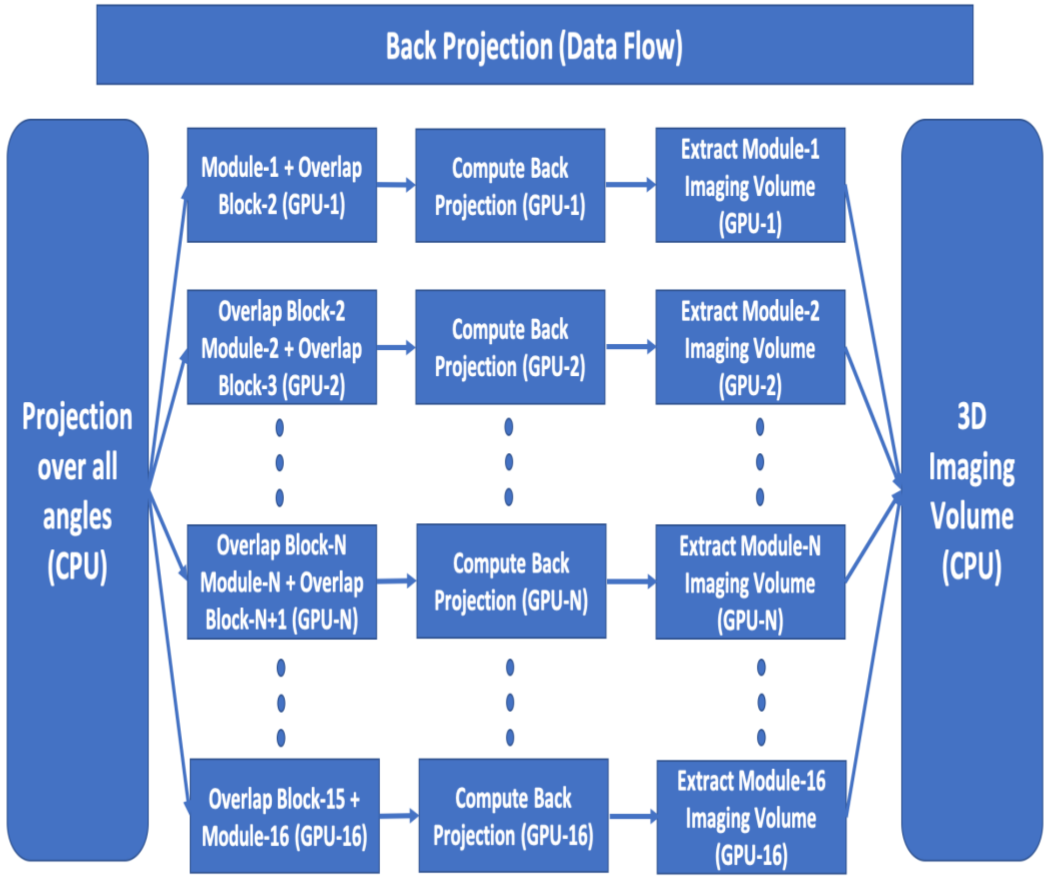

Supplementary Fig. S2: Data Flow from CPU to multi-GPU while computing the back projection
